# Supplementary material for: Superconductivity in the antiperovskite Dirac-metal oxide Sr3−xSnO
Source: Nat Commun. 2016 Dec 12;7:13617. doi: 10.1038/ncomms13617 (PMC5159807; doi:10.1038/ncomms13617)
Supplement: Supplementary Information — Supplementary Figures 1-5 and Supplementary References. [file ncomms13617-s1.pdf]

Supplementary Figures

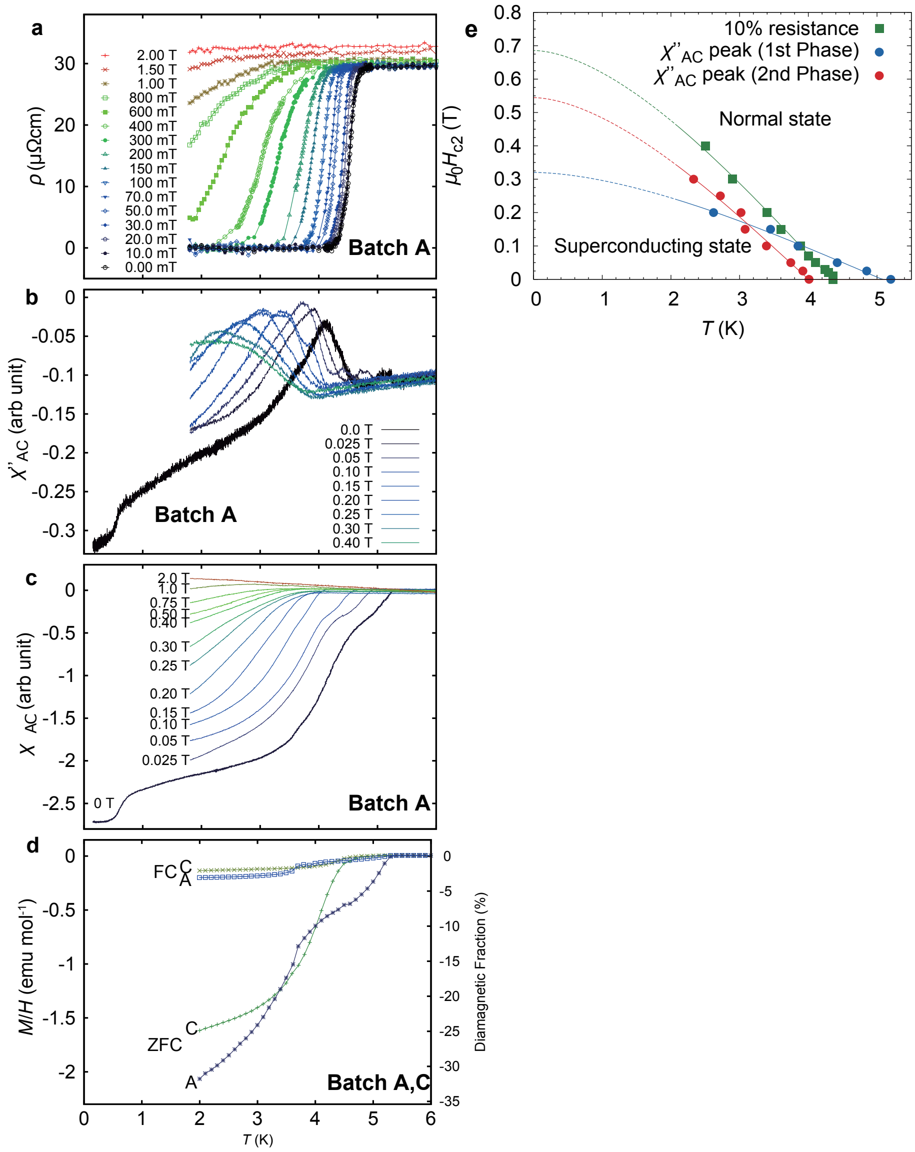

Supplementary Figure 1 | **Superconducting properties of samples from different batches. a,** Resistivity ρ under zero and various magnetic fields for a batch A sample. **b, c,** Imaginary and real parts of AC susceptibility $\chi'_{\rm AC}$ and $\chi''_{\rm AC}$ for batch A sample. **d,** Magnetization under zero-field-cooling (ZFC) and field-cooling (FC) and with applied field of 0.5 mT for samples from batches A and C. The vertical scale on the right indicates the estimated diamagnetic volume fraction. The shoulder at around 3.7 K seen in the curve for the batch A is due to pure Sn contaminating the sample. **e,** Field-temperature phase diagram of superconductivity in $Sr_{3-x}SnO$ for a batch-A sample. The upper critical field H_{c2} is extracted from both the 10% resistivity and the two transition features in AC susceptibility. The curves are results of fitting with the Wertheimer-Helfand-Hohenberg relation[1].

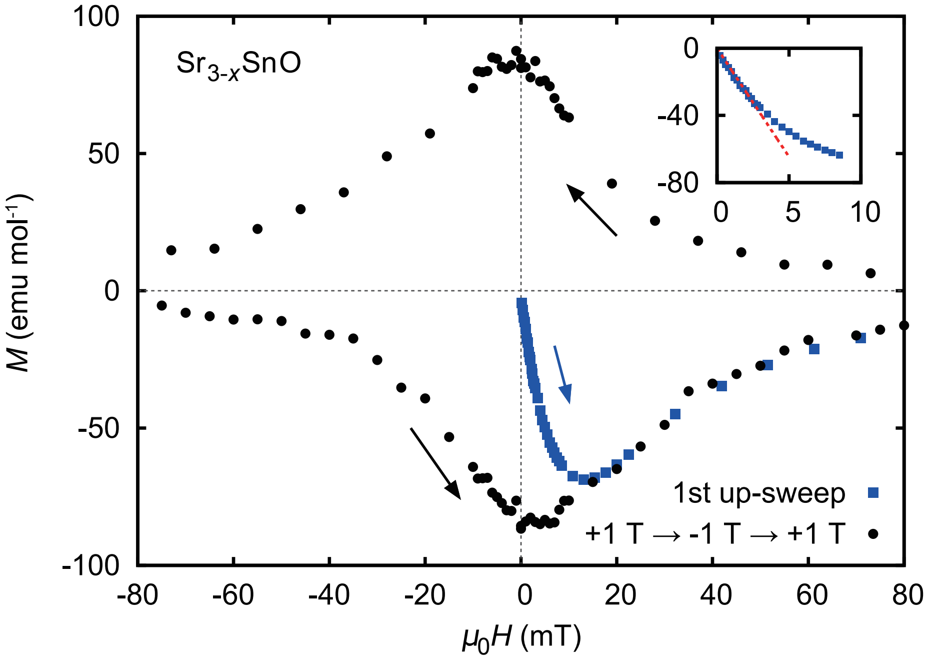

Supplementary Figure 2 | **Magnetization curve of a $Sr_{3-x}SnO$ sample at 2 K.** The curve was obtained for a batch-C sample in a field sweep loop: 0 T → 1 T → -1 T → 1 T. The inset shows a linear fitting to the initial up-sweep curve at low field (red line). Deviation from linear behaviour is used to estimate the lower critical field H_{c1} to be about 2.4 mT.

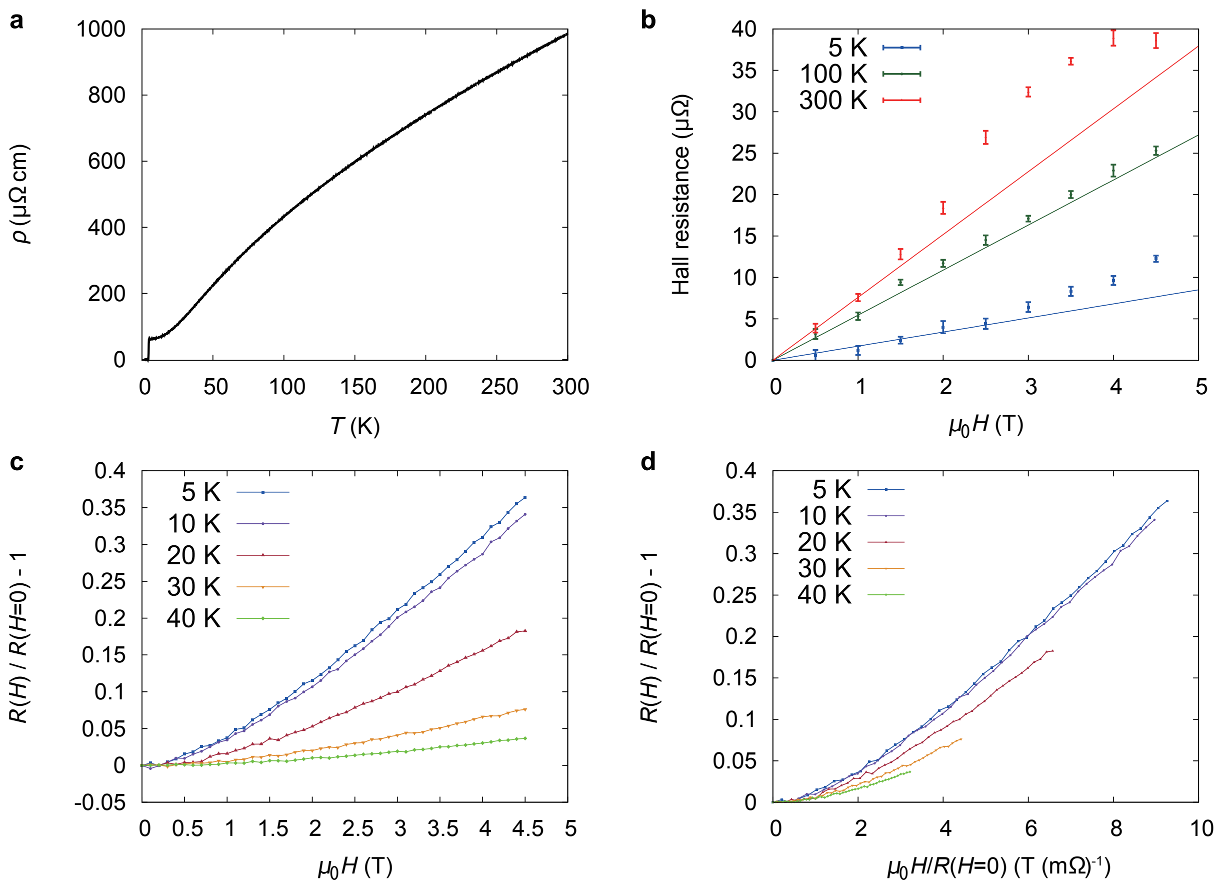

Supplementary Figure 3 | **Transport properties of $Sr_{3-x}SnO$. a,** Resistivity between 1.8 and 300 K for a batch-C sample. **b,** Hall resistance as a function of applied field at various temperatures, indicating hole-like carriers for a batch-A sample. Each error bar indicates the standard error of five data acquired under a same condition. **c,** Transverse magnetoresistance at various temperatures for a batch-A sample. **d,** Kohler plot of normalized magnetoresistance vs. the magnetic field divided by the zero-field resistance for a batch-A sample.

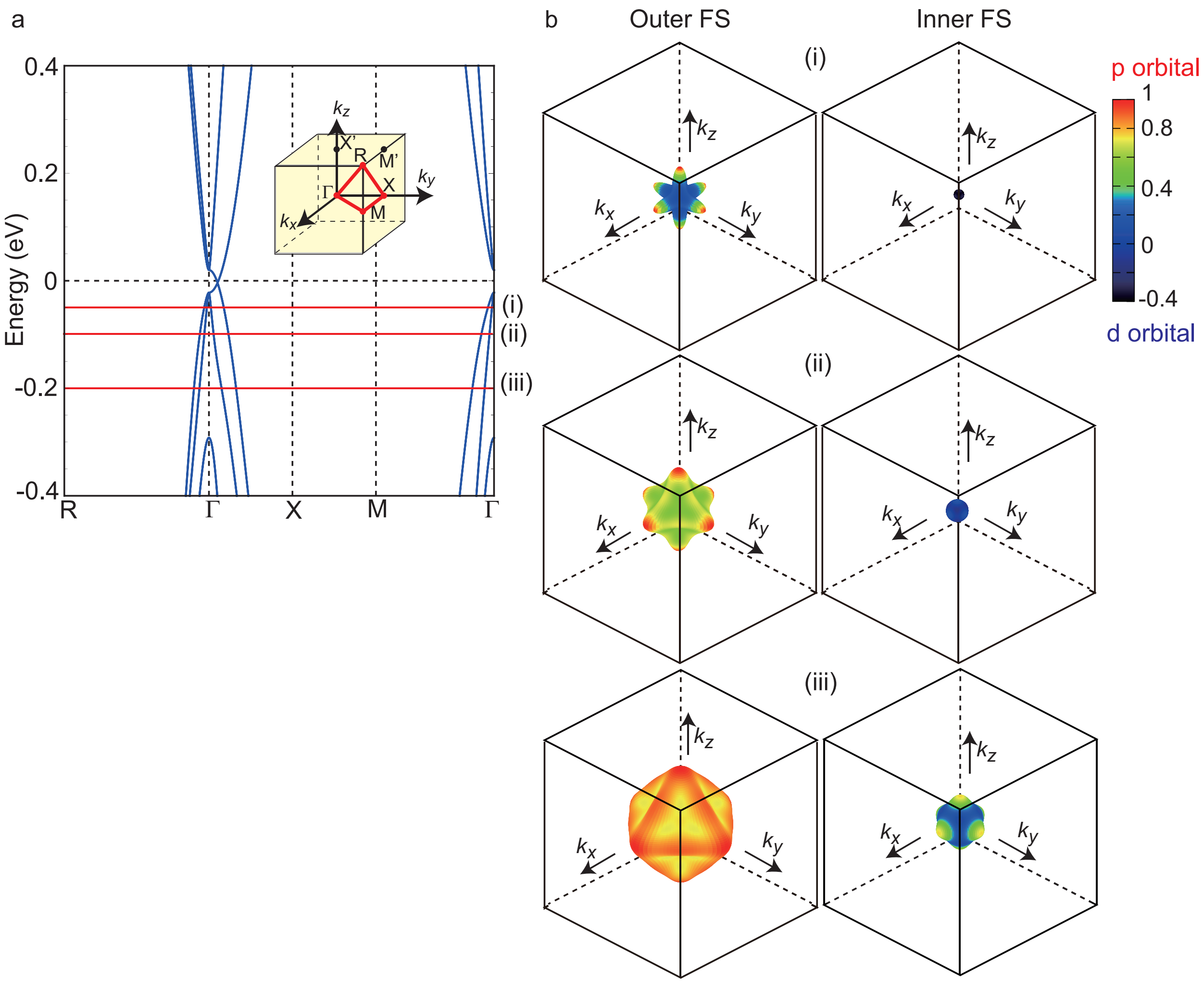

Supplementary Figure 4 | **Fermi surface (FS) evolution and orbital texture.** **a,** Tight-binding band structure with the red lines indicating the Fermi levels at (i) -0.05 eV, (ii) -0.1 eV, and (iii) -0.2 eV. **b,** Orbital texture on the outer and inner FSs around the Γ point from the tight-binding calculation with the Fermi levels for (i), (ii), and (iii). The colour represents the degree of p-d orbital mixing, $\left(|\psi_p|^2 - |\psi_d|^2\right) / \left(|\psi_p|^2 + |\psi_d|^2\right)$. Here, ψ_p and ψ_d are Sn-5p and Sr-4d orbital wavefunctions. The orbital mixing becomes stronger near the Dirac point.

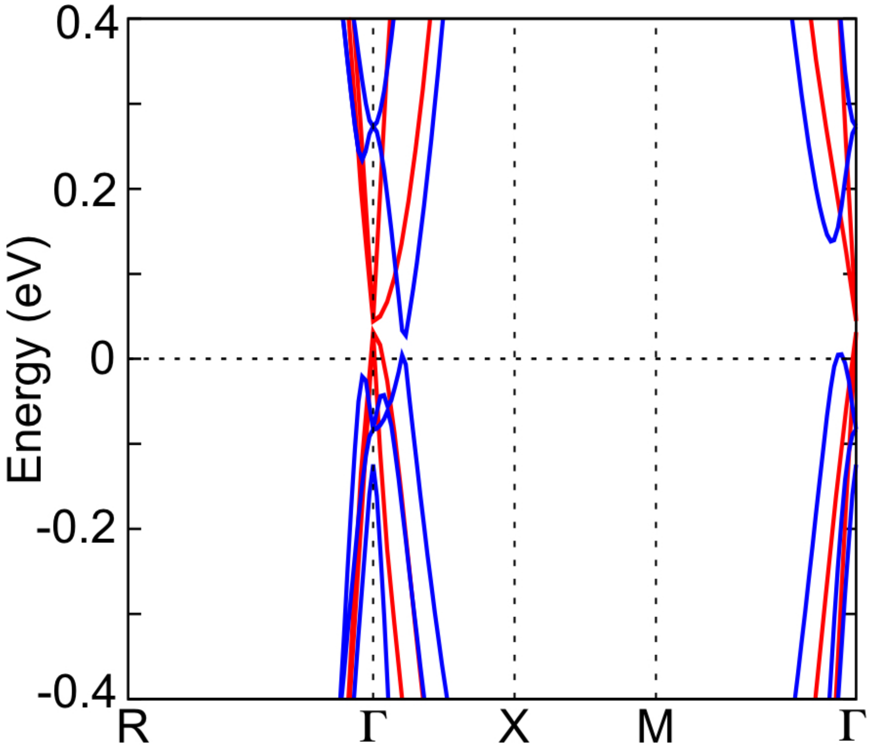

Supplementary Figure 5 | Band structures of Sr_3SnO in the first principles calculations. These band structures are obtained using the WIEN2k package[2] (blue curves) and the Vienna ab-initio simulation package (VASP)[3–5] (red curves). Spin-orbit coupling is included in both calculations. For the calculation with the WIEN2k package, the Perdew-Burke-Erzerhof generalized-gradient approximation[6] is used for exchange-correlation function. The parameter $(RK_{\rm max},\ G_{\rm max}\ )=\ (9.0, 14.0)$[7] are chosen, and 20 $\times$ 20 $\times$ 20 momentum points in the irreducible Brillouin zone are used in the self-consistent cycle. The crystal parameters are taken from the experimental results[8]. The radii of the spherical atomic regions for Sr, Sn, O are chosen to be 2.33, 2.50, and 2.33 (a.u.), respectively. In the band structure obtained with the VASP, the Heyd-Scuseria-Ernzerhof (HSE) screened Coulomb hybrid density functionals[9] are employed to improve the underestimation of band gap. Besides, the projector augmented-wave potential[10,11] and the 5 $\times$ 5 $\times$ 5 Monkhorst-Pack grid are used. The energy cutoff of the plane wave basis is 400 eV, and we performed structural relaxations with forces converged to less than 0.001 eV/Å. We note that for Sr_3SnO the band spectrum generated using the WIEN2k package is very different from that in ref. 5, where the HSE screened Coulomb hybrid density functionals were employed in the first principles calculation. First, details of the band structure near the Γ point are substantially different. Second, there is no hole Fermi surface (FS) around the R-point, whereas it is present in the result of ref.5. In contrast, the band spectrum generated using the VASP with the HSE method almost reproduces the band spectrum in ref. 5 near the Γ point, despite that there is no hole FS around the R-point again. Such method-dependence was discussed before by Vidal *et al.*[12], which concludes that the HSE method is more reliable. Thus we adopt the HSE result to parametrize our tight-binding band structure near the Γ point to obtain the orbital texture (Fig. 4 and Supplementary Fig. 4).

Supplementary References

1. Werthamer, N., Helfand, E. & Hohenberg, P. Temperature and purity dependence of the superconducting critical field, H_{c2}. III. Electron spin and spin-orbit effects. *Phys. Rev.* **147**, 295–302 (1966).

2. Blaha, P., Schwarz, K., Madsen, G., Kvasnicka, D. & Luitz, J. WIEN2k. *An Augmented Plane Wave and Local Orbitals Program for Calculating Crystal Properties* (2001).

3. Kresse, G. & Hafner, J. Ab initio molecular dynamics for open-shell transition metals. *Phys. Rev. B* **48**, 13115 (1993).

4. Kresse, G. & Furthmüller, J. Efficiency of ab-initio total energy calculations for metals and semiconductors using a plane-wave basis set. *Comput. Mater. Sci.* **6**, 15–50 (1996).

5. Kresse, G. & Furthmüller, J. Efficient iterative schemes for ab initio total-energy calculations using a plane-wave basis set. *Phys. Rev. B* **54**, 11169 (1996).

6. Perdew, J. P., Burke, K. & Ernzerhof, M. Generalized gradient approximation made simple. *Phys. Rev. Lett.* **77**, 3865 (1996).

7. Madsen, G. K., Blaha, P., Schwarz, K., Sjöstedt, E. & Nordström, L. Efficient linearization of the augmented plane-wave method. *Phys. Rev. B* **64**, 195134 (2001).

8. Nuss, J., Mühle, C., Hayama, K., Abdolazimi, V. & Takagi, H. Tilting structures in inverse perovskites, M_3TtO (M= Ca, Sr, Ba, Eu; Tt= Si, Ge, Sn, Pb). *Acta Cryst. B* **71**, 300–312 (2015).

9. Krukau, A. V., Vydrov, O. A., Izmaylov, A. F. & Scuseria, G. E. Influence of the exchange screening parameter on the performance of screened hybrid functionals. *J. Chem. Phys* **125**, 224106 (2006).

10. Blöchl, P. E. Projector augmented-wave method. *Phys. Rev. B* **50**, 17953 (1994).

11. Kresse, G. & Joubert, D. From ultrasoft pseudopotentials to the projector augmented-wave method. *Phys. Rev. B* **59**, 1758 (1999).

12. Vidal, J., Zhang, X., Yu, L., Luo, J.-W. & Zunger, A. False-positive and false-negative assignments of topological insulators in density functional theory and hybrids. *Phys. Rev. B* **84**, 041109 (2011).
